# Research on urban three-dimensional greening design from the perspective of climate change—a case study of Beilin District, Xi’an, Shaanxi Province, China

**DOI:** 10.1007/s11356-023-31386-8

**Published:** 2023-12-26

**Authors:** Wei Wang, Jinbang Zhang, Jiaying Li

**Affiliations:** Art School, Xi’an University of Architecture and Technology, No. 17, Yanta North Road, Beilin District, Xi’an, 710005 Shaanxi China

**Keywords:** Climate change, Greenery, Space, Ecology, Streets, Future design

## Abstract

Climate change is an important issue for cities today and in the future. At present, China has a large population and complex climate conditions, and cities are also vulnerable to the adverse effects of climate change (Tian, Environ Sustain Dev 6: 153-155 2020). Three-dimensional greening can not only improve the green space system of a city but also have a far-reaching impact on the ecology, image, and economic benefits of a city. Therefore, the study of urban three-dimensional greening is an effective means to deal with climate change strategies. By exploring the influence of traditional greening and three-dimensional greening on Local Climate in Beilin District of Xi’an, Shaanxi Province, the mechanism of three-dimensional greening on urban ecological environment was discussed, and the ecological theory, urban three-dimensional greening theory, and urban local climate zone (LCZ) were referred to. Based on the methods of national climate monitoring, ENVI-met simulation, and field independent measurement, this paper selected a research sample site in the east section of Jianshe Road, Beilin District, Xi’an City, Shaanxi Province, China, and applied ENVI-met software to simulate the thermal stress relationship among building exterior surfaces, plants, and air in the street; quantified the overall ecology of the area; and used measuring instruments. The influence of different types of greening in the base on the site temperature, humidity, CO_2_ (carbon dioxide) concentration, wind speed, and other climate factors data was, respectively, measured and analyzed. The grid analysis was used to compare the traditional greening and three-dimensional greening, then the numerical differences of each impact factor were sorted out, and the effect of three-dimensional greening on the improvement of urban ecological environment was discussed by analyzing the climate factors with greater impact. The results show that (1) three-dimensional greening plus traditional greening is the most beneficial mode; (2) in the same environment, according to the parameter of 1.5 m from the ground in the model environment, it can be seen that the temperature of the space treated with three-dimensional greening of buildings is reduced by 3.5–3.6 ℃ compared with the control group, the relative humidity is different by 7–8%, the CO_2_ concentration is reduced by about 5%, and the spatial wind speed is relatively small. (3) When the urban green coverage rate is more than 40%, the improvement of temperature is more obvious, if it reaches 50%, the cool phenomenon in summer can be fundamentally changed. From the perspective of human perception, the PMV index increased by 0.27 on average. This paper discusses and analyzes the three-dimensional greening of urban streets in Beilin District, Xi’an City, Shaanxi Province, China, and studies its influence on urban ecology to different degrees. The conclusions are as follows: Different types of greening have different degrees of influence on urban climate. Meanwhile, the experimental results of this paper show that in cities like Xi’an, Shaanxi Province, China, where summer is hot, adding three-dimensional greening to traditional street greening can significantly improve the environmental microclimate, which is an effective means to cope with climate change, improve the site environment, and stabilize the urban ecosystem.

## Introduction

**Fig. 1:**
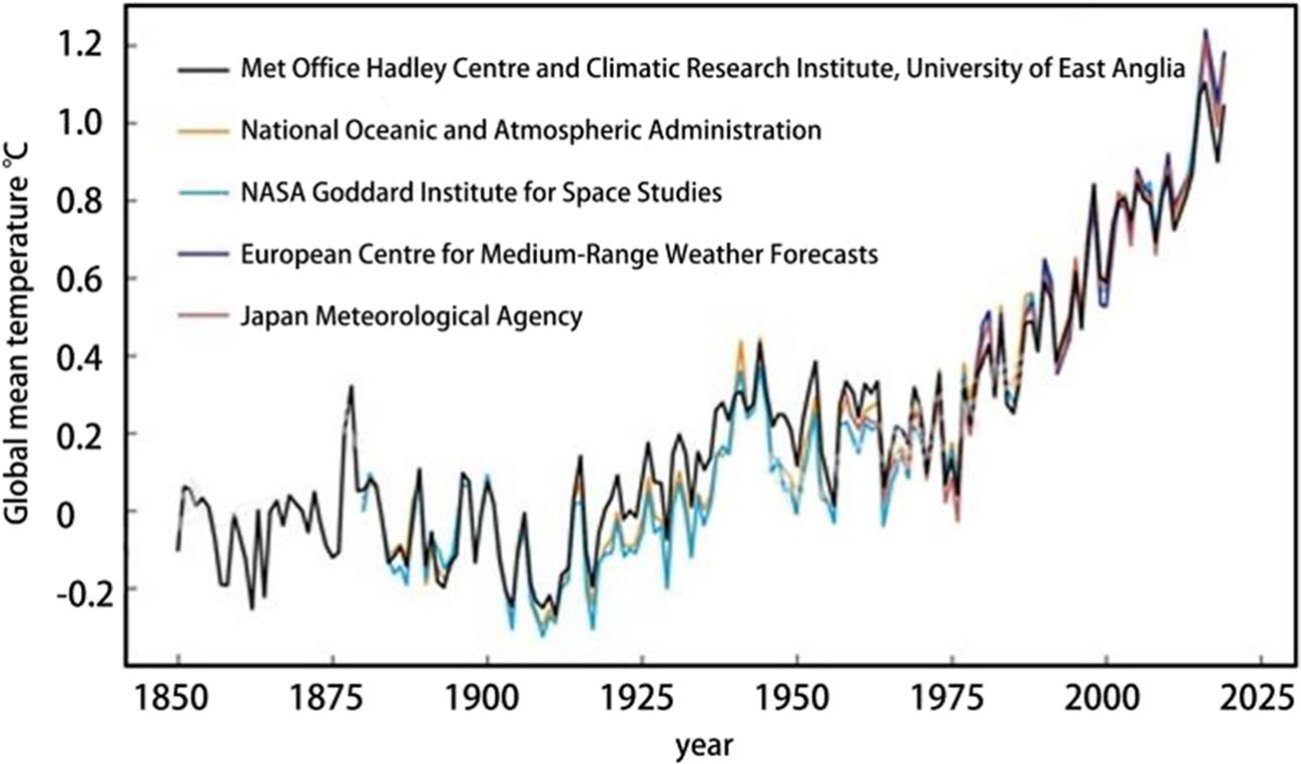
Global mean temperature anomalies from 1850 to 2019 (relative to the 1850–1900 average)

Global warming is a major environmental problem facing the world today. With the continuous increase of human activities, a large amount of greenhouse gas emissions such as carbon dioxide lead to changes in the earth’s climate, and then cause global warming (Bruce 2023). At present, the global warming trend is still accelerating rapidly. In 2019, the global average temperature was about 1.1 °C above pre-industrial levels, making it the second-warmest year on record for complete meteorological observations. Climate change in China is quite consistent with global climate change, but there are also clear differences (Gui et al. 2022). In the past hundred years, China’s annual average surface temperature has increased significantly, with the rate of warming slightly higher than the global average in the same period, and annual CO_2_ emissions have also shown an increasing trend (Zhu et al. 2019). In terms of precipitation, the trend of precipitation change in the last 100 years and the last 50 years is not obvious. According to the current situation of China’s response to climate change, in the next 100 years, China’s surface temperature will continue to rise, and precipitation will also show an increasing trend (Fig. 1), both of which play an important role in the process of responding to climate change. In order to alleviate the current situation, restoring the balance of greenhouse gases has become the key to controlling climate warming.

In the case of shrinking urban green space and deteriorating climate, it is the development direction of modern urban construction to explore an optimal path to effectively use the limited urban space, improve the urban climate, and greatly increase the area of green space (Reynolds et al. 2019; Alexandru et al. 2022). In a particular climatic zone, which vertical greening mode performs best in a complete cycle, and what is the appropriate vertical greening mode for each climatic zone, these problems become the key problems to be solved in this study.

At present, most of the research on three-dimensional greening in China is theoretical, and the practical projects are relatively few, and they are rarely applied to urban landscape. Most of the research still stays in the traditional plant design principles, as well as the simple design presentation of plants, and some studies in greening technology, management and maintenance, theoretical methods, etc., but most of them simply put forward some suggestions for improvement, and did not carry out detailed discussions on these suggestions, nor did they go into the specific implementation steps. Beilin District is the area with the largest coverage in Xi’an, Shaanxi Province, China. To investigate, analyze, and summarize it not only has certain research significance but also can make some practical contributions for Xi’an and cities in different countries with different climates or climate zones to determine the best vertical greening model and quantify the improvement effect of three-dimensional greening on environment and climate. At present, ecological city is the direction of future construction and development. The increase of urban green area and the construction of energy-saving and low-carbon cities have become the general requirements of society. Therefore, combining the ecological concept with three-dimensional greening and putting forward relevant suggestions and practical means for the landscape needs of modern urban streets is of great practical significance for studying the unique climate characteristics of the site and creating the landscape form of urban streets under the theory of ecology.

## Methodology

### Study area

**Fig. 2:**
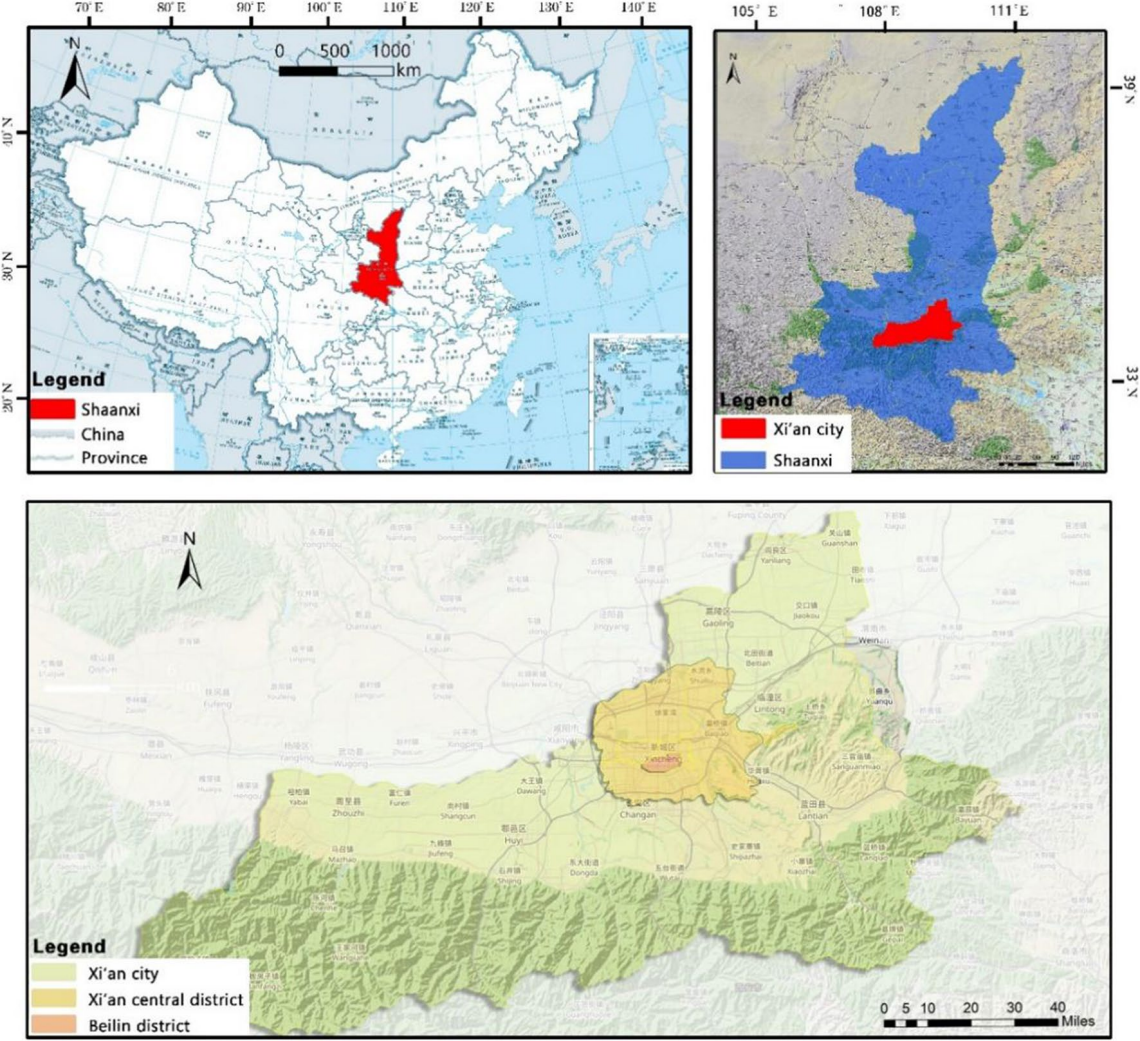
Schematic diagram of the study area

**Fig. 3:**
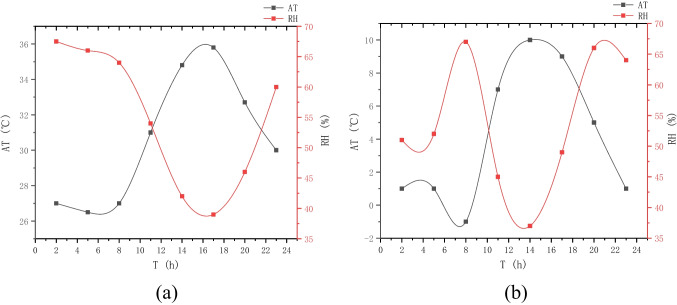
Distribution of typical meteorological daily air temperature and relative humidity in Xi‘an: **a** summer; **b** winter

**Fig. 4:**
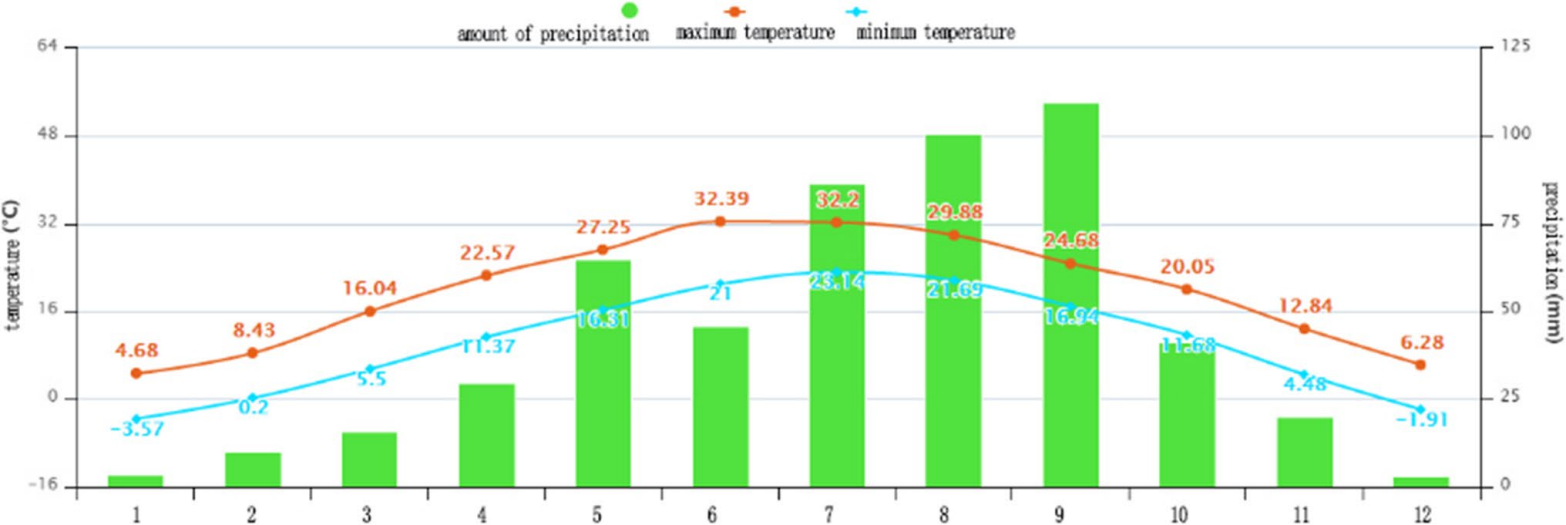
Monthly mean temperature and precipitation in Xi’an from 2005 to 2015

The main research area of this paper is Beilin District, Xi’an City, Shaanxi Province, China (Fig. 2), which is located in the southeast of Xi’an and covers an administrative area of 23.37 km^2^. In summer, Xi’an has a serious heat wave in summer, and the temperature in the city is obviously higher than that in the surrounding counties, and the urban heat island effect is obvious. The annual average temperature is 13–17 ℃. January is the coldest, with an average temperature of − 0.9 to 1.7 ℃, while July is the hottest, with an average monthly temperature of more than 25 ℃ (Fig. 3). The northeast wind prevails throughout the year, with an average annual wind speed of 1.6 m/s. The annual precipitation is 522.4–719.5 mm, gradually increasing from north to south, and July and September are the peak precipitation months (Fig. 4).

**Fig. 5:**
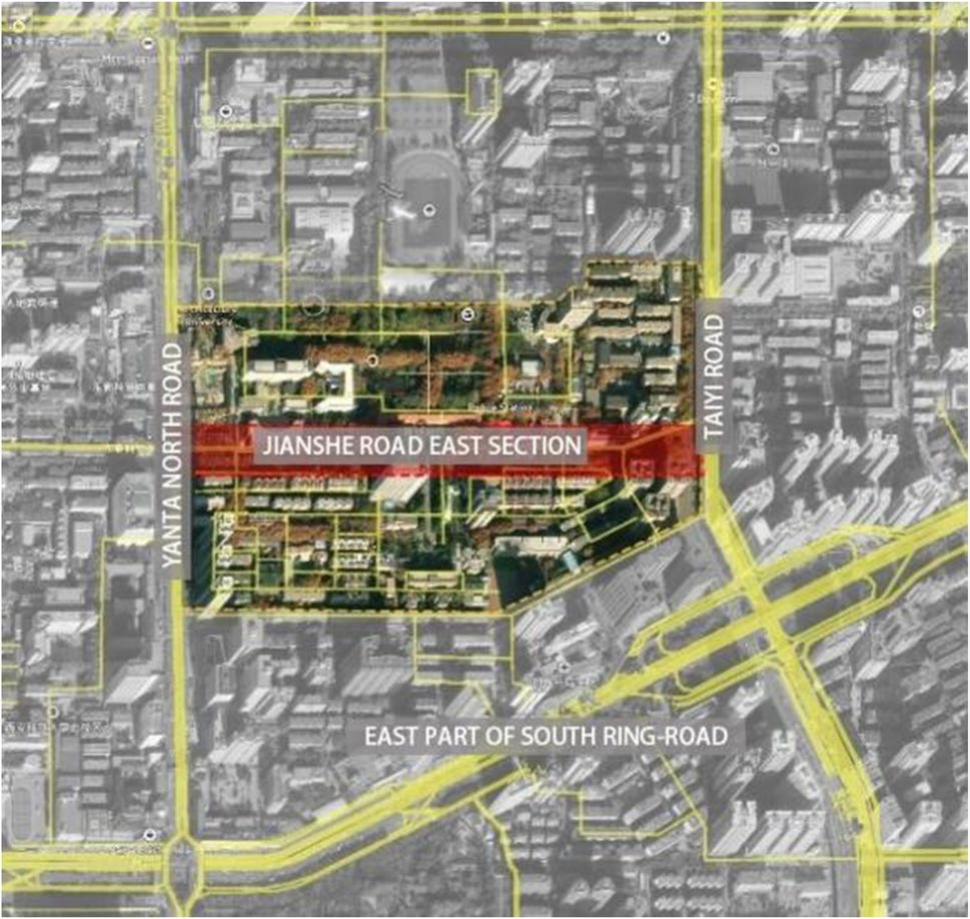
Location analysis map of east section of Jianshe Road

**Fig. 6:**
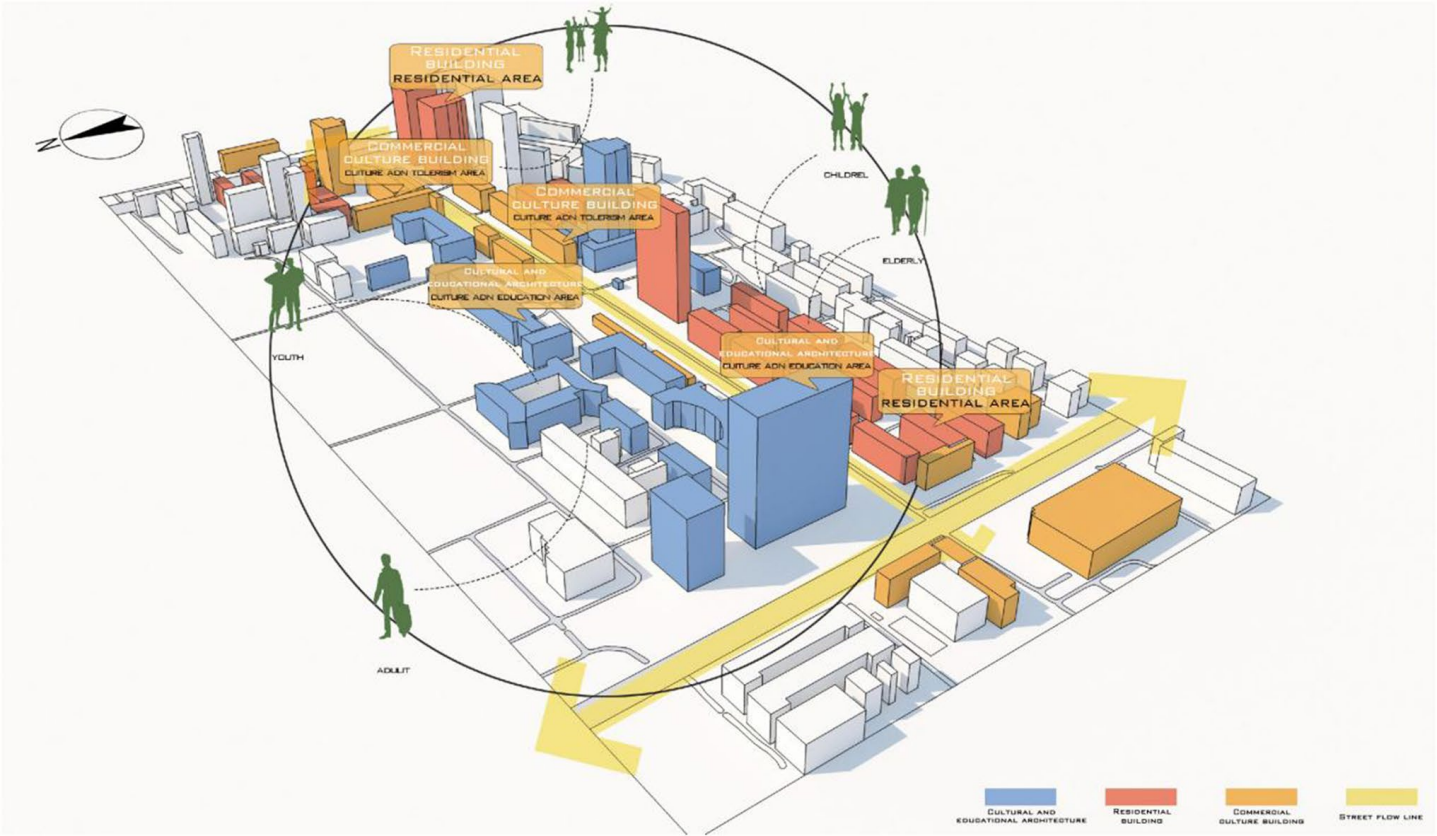
Overall analysis of east section of Jianshe Road

This section focuses on the analysis of the east section of Jianshe Road, Beilin District, Xi’an, Shaanxi Province, located in the southeast of Beilin District, south of Xi’an City (Fig. 5), adjacent to the north and south campuses of Xi’an University of Architecture and Technology, small supermarkets, night city, all kinds of commercial complexes, close to the subway. It is adjacent to Taiyi Road in the south and Yanta North Road in the north (Fig. 6).

### Data collection

To scientifically quantify the impact of vertical greening patterns, the main analytical tool of this study was the ENVI-MET analytical model, which is commonly used to calculate energy transfer between buildings, vegetation, ground, air, and thermal comfort (Bruno and Yin 2021). Unlike other fluid dynamics simulation software, the Enviro-MET model incorporates thermodynamic principles and has the unique capability of analyzing the reflection and absorption of short- and long-wave radiation by plants (Sun et al. 2021). As such, it is the analytical tool of choice for researchers. The Reynolds average Navier–Stokes equation for the Environment-MET is given below.

### Climate change analysis

#### Land surface temperature inversion

By remote sensing images and the method of combining, the classification of urban greening in downtown Xi’an six administrative areas, using remote sensing images and related products through the temperature vegetation index method, surface temperature inversion for Xi’an urban area near raster data, on the basis of the analysis the influence of different urban vertical greening on the surface temperature mechanism.

Landsat is a series of satellites launched by NASA, and 8 satellites have been launched so far. The Landsat series data can be downloaded free of charge by registering on the US Geological Survey (USGS) website. In this study, the land surface temperature inversion was performed using the mono-window algorithm to simulate the urban thermal environment of the planning area. The heat island effect of the planning area is analyzed through the inversion temperature results, which provides powerful technical support for urban design. It includes three main calculation steps: image radiometric calibration, surface emissivity calculation, blackbody radiance, and surface temperature calculation.

To eliminate the deviation in the imaging process of different remote sensing images, data fusion processing is required when calculating LST in each region. Firstly, the radiation and atmospheric correction data of remote sensing images are preprocessed. In this paper, surface temperature inversion is performed based on the radiation transfer equation method, and then calculate the urban heat island intensity, and the formula is as follows:

1 $${\text{B}}({\text{LST}})=({L}_{\lambda }-{L}_{\uparrow }-\tau \times (1-\varepsilon )\times {L}_{\downarrow })/ (\varepsilon \times \tau )$$

2 $${\text{LST}}={\mathrm{ K}}_{2}/{\text{ln}}({{\text{K}}}_{1}/{\text{B}}({\text{LST}})+1)$$

3 $${{\text{UHII}}}_{{\text{i}}}={{\text{LST}}}_{{\text{i}}}-{{\text{LST}}}_{{\text{p}}}$$

In the formula, *B*(LST) is the radiation brightness value of the blackbody in the thermal infrared band. *L*_λ_ is the on-star radiance; The parameters *L*_↑_, *L*_↓_, and *τ ε* represent atmospheric upward radiation, atmospheric downward radiation, and atmospheric transmittance, respectively. *K*_1_ and *K*_2_ can be obtained from the image header file. *ε* is the surface specific emissivity and is an important parameter for temperature inversion. LST_i_ represents the average surface temperature in the study area; LST_p_ represents the average surface temperature in vegetated areas, and the difference is used as urban heat island intensity (UHIIi) of the block (the vegetation area obtained by NDVI index).

#### ENVI-met simulation

**Fig. 7:**
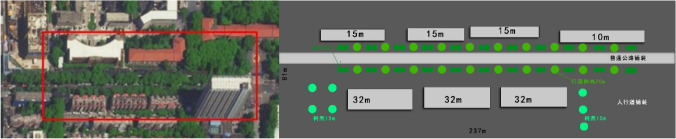
The simulated floor plan was drawn according to the image map of the simulated area sample

The 237-m street in the east section of Jianshe Road in Beilin District of Xi’an City was selected as the research base. Measuring instruments were used to measure the temperature, humidity, CO_2_ concentration, and other influencing factors in different areas of the research base. The images from Google Earth were imported into AutoCAD to draw the base map of teaching buildings, which was saved as BMP format and used as the modeling basis in ENVI-MET. The longitude, latitude, and model size were set in Spaces, and the BMP base map was imported to draw the building, vegetation, underlying surface, and other elements successively. The underlying surface types include soil (loamy), stone pavement (basalt brick), granite pavement (granit shining), and asphalt road (Fig. 7).

The building height was set according to the building height measured in the field. Different simple plants and 3D plants were matched in the model according to the vegetation species, height, crown width, and other information in the field survey. In the Albero module, a 3D plant model can be created. In order to improve the model accuracy, leaf area density (LAD) of five gradients was set according to the actual 3D green amount. It should be noted that the center of the 3D plant needs to leave a blank space (Table 1). Save the model as an INX file. Next, set the simulation date, start time, and simulation duration in EnVI-Guide. Select intermediate mode, set wind speed to 2.5 m/s, wind direction to NE45, temperature range from 14 to 26 °C, humidity range from 20 to 60%, and save the file as SIMX file. Start the ENVI-Core module, load the model, and start the simulation.

**Table 1:** Simulation parameters

type	project	parameter
	Simulation date	2020/06/13
	The simulation time	14:00–15:00 (1H)
	The grid number	50*50*40
	Accuracy of the grid	5*5*3
model	Geographical coordinates	E: 108.96
		N: 34.23
	Stress pattern	Simple forcing

### Climate change simulation

#### Simulation of climate change under traditional green coverage

Among the five types of underlying surfaces in natural environments studied in this study, the air temperature of the underlying surfaces in natural environments was significantly lower than that of the underlying surfaces in built-up areas, except for UUS B (sparse woodland) and UUS F (hard pavement). For the mean air temperature data in the morning, the air temperature distribution of each underlying surface type generally showed the following patterns: UUS F (hard pavement, 34.33 ℃) > UUS B (sparse woodland, 34.04 ℃) > UUS D (natural bare land, 33.79 ℃) > UUS C (low vegetation, 34.04 ℃) 33.23 ℃ > UUS A (dense woodland, 33.02 ℃) (Table 2). The hard pavement and natural bare ground absorb a large amount of solar radiation because the space is open and unobtrusive. At the same time, the soil, sand, and stone in the underlying surface of the bare ground and the cement and concrete on the underlying surface of the hard pavement have strong heat storage capacity, which leads to more radiant heat and higher temperature in the space. The hard paved area also increases in temperature due to the heat release of road traffic vehicles and a series of human activities (Zhai et al. 2021). Compared with the underlying surface of the two types of natural environment areas, the underlying surface of vegetation type provides water vapor due to transpiration of vegetation leaves, and increases air humidity, resulting in lower air temperature (Xu et al. 2018; Xiao and Ji 2020). Among the three types of vegetation types, the dense forest area has the lowest air temperature. This is because the dense forest area has higher vegetation height and coverage, which can effectively shield the solar radiation and have larger vegetation leaf area, thereby enhancing transpiration and significantly reducing air temperature. The temperature of sparse forest area is related to the complex land cover property in the region. Apart from sparse trees, there are also some bare land areas in the sparse forest area, resulting in the highest air temperature. The reason why the air temperature in low-rise vegetation areas is higher is that the areas are all open Spaces, which can receive a large amount of solar radiation without radiation shielding. Although plant leaves can reduce the space temperature through transpiration, the large amount of radiation heat absorbed by the open space cannot be compensated by the relatively weak transpiration and cooling effect of low-rise vegetation, so the temperature is higher.

**Table 2:**
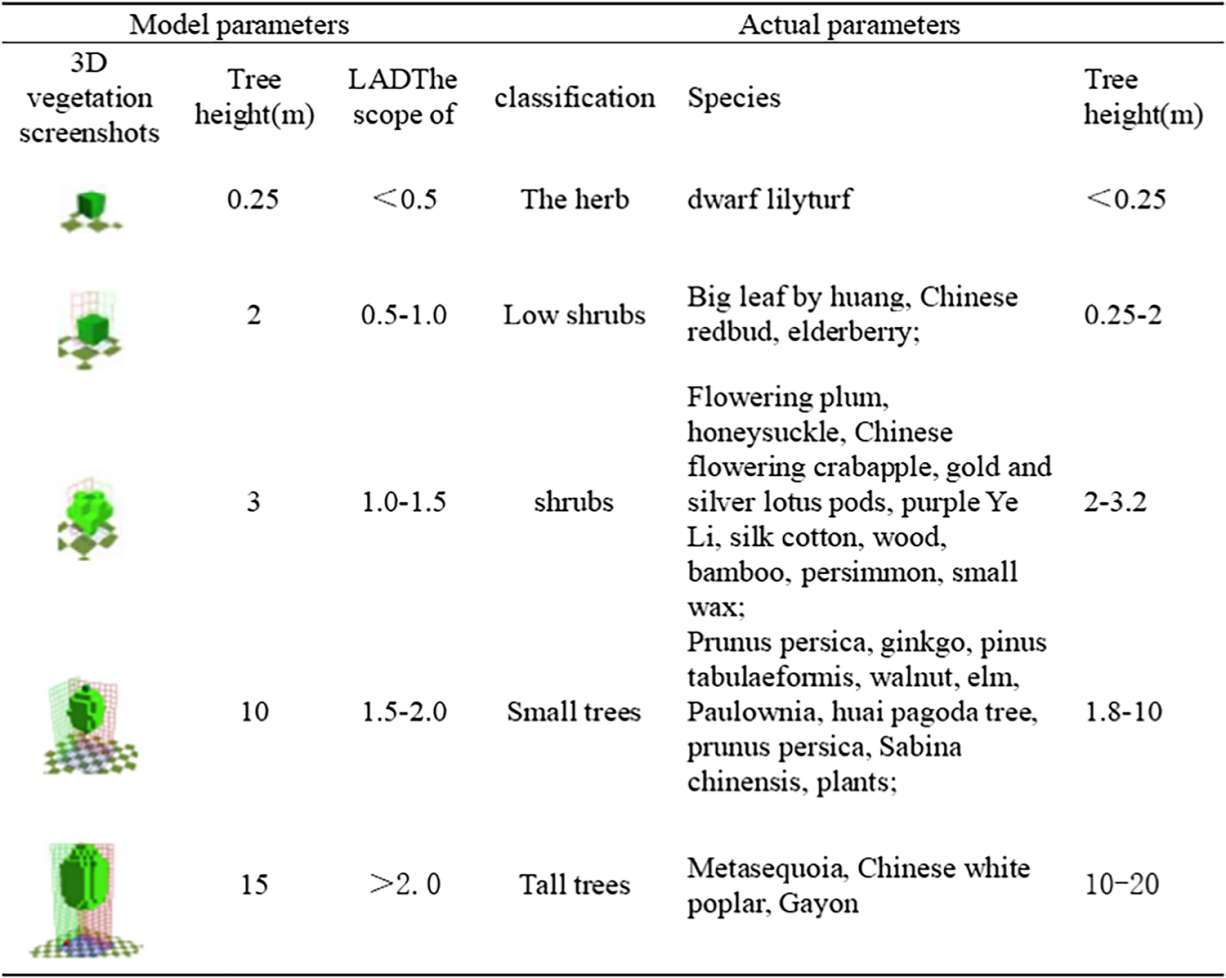
Green Factors in the Model

**Fig. 8:**
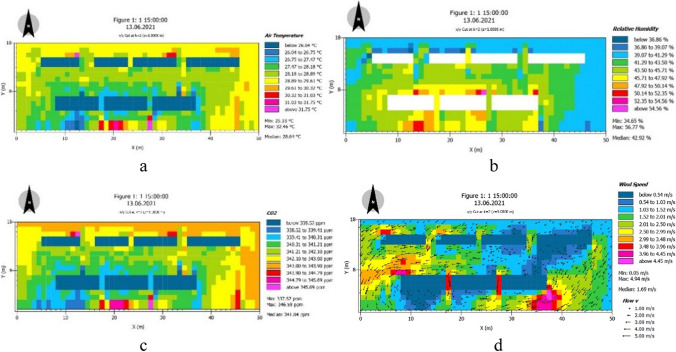
Climate simulation of traditional greening 2 m away from the ground: (**a**) air temperature; (**b**) humidity; (**c**) CO_2_ concentration; (**d**) wind speed

The vegetation types of urban green space can be generally divided into three categories, including trees, shrubs, and grasslands. Depending on the size of green space, there may be a variety of configurations (Mahoro et al. 2022). Large- and medium-sized green spaces generally contain three combinations of tree, shrub, and grass, with complex structure and high species richness, while small green spaces have a relatively simple vegetation structure, and their corresponding ecological and environmental effects are insufficient. In addition, the dense land use classification in urban space also leads to the shortage of large-scale green space and the scattered layout of small- and medium-sized green space (Liu 2021). Therefore, it is of great significance to study the allocation of green space on the microscale for how to better exert the ecological and environmental effects (Hua et al. 2022). The three-dimensional microclimate simulation software ENVI-Met was used to set four groups of green spaces with different configurations under ideal conditions, namely, (1) trees + shrubs + grass, (2) trees + shrubs, (3) trees + grass, and (4) grass. The four groups of green spaces had different three-dimensional green ratio and green coverage, and the different weather performances of these four groups of green spaces under the same scenario were analyzed. The basic parameters of the model remained the same, and the initial meteorological parameters remained unchanged. The simulation period was September 1, 2020, with a start time of 7:00 and a duration of 2 h. Meteorological parameters included wind speed 2.5 m/s, wind direction 0° north, temperature range of 18–28 ℃. The vegetation was three simple plants: grassland (0100XX, height 0.5 m), shrub (0000H2, height 2 m), tree (0000SK, height 15 m). The grid of 1 * 1 * 1 was defined as the three-dimensional green quantity A, and the three-dimensional green quantity of each tree and shrub was 30A, 4A, and A, respectively. The boundary conditions were simple forcing (force). In order to exclude other influencing factors, the surrounding urban environment was not set in this experiment, and only a simple vegetation model was set in a certain size of open space for simulation test (Fig. 8).

#### Simulation of climate change under three-dimensional green coverage area

**Fig. 9:**
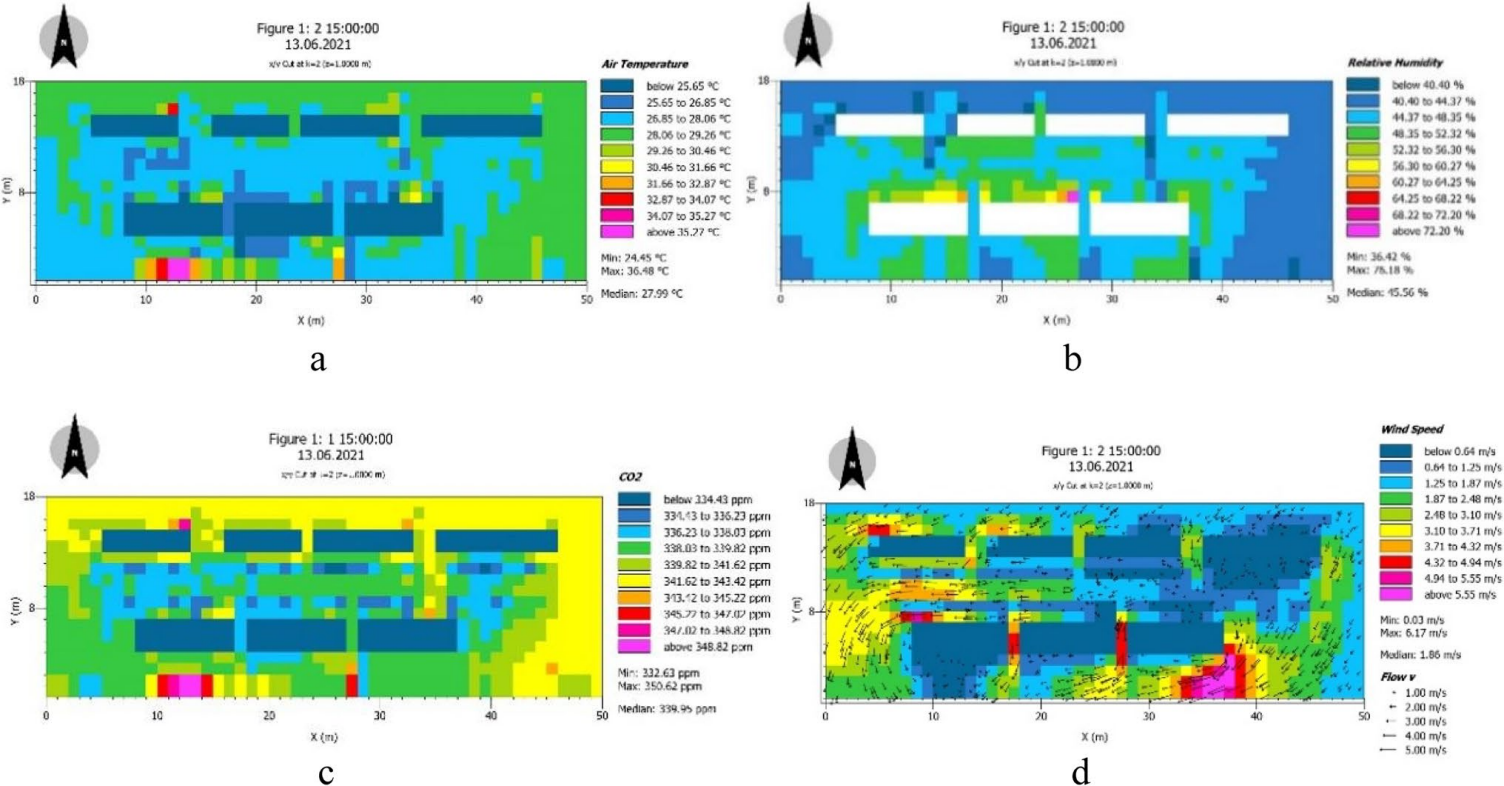
Climate simulation map of three-dimensional greening 2 m away from the ground: (**a**) air temperature; (**b**) humidity; (**c**) CO_2_ concentration; (**d**) wind speed

The microclimate of the city and the energy exchange process of the building facade constitute an integrated system that is constantly interacting (Washim et al. 2023). ENVI-met models heat and water vapor transfer from the exterior walls of buildings at high resolution and makes predictions of wall and interior temperatures (Li and Zhong 2021). Vegetation and hydrological characteristics are some of the key factors in optimizing urban thermal comfort. ENVI-met provides a sophisticated vegetation model that can simulate transpiration, CO_2_ uptake, and leaf temperature based on the photosynthetic rate of the plant, and can simulate the wall and roof system of the vegetation, as well as the effects of different growing media and installation methods including the plant (Chen and Liu 2020). ENVI-met also includes a separate water drop dispersion and evaporation model that simulates the cooling effect of fine water spray on air temperature. On the basis of the above, this paper simulates the external walls of buildings with roof greening and vertical greening. The effects of various growth media and planting methods containing plants were simulated (Fig. 9).

Investigation and research on people’s perception of climate change in vertical planting coverage area.

**Fig. 10:**
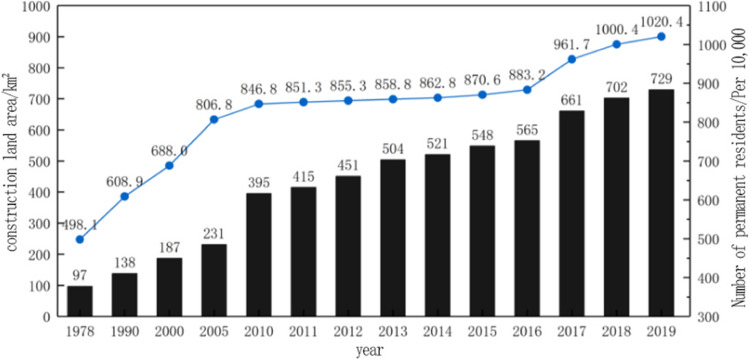
Map of population survey data

The main survey method was random survey in urban crowded areas, and a total of 136 valid questionnaires were collected. Among them, 53.8% and 46.2% were in the covered area and the uncovered area, respectively. Males accounted for 51.8% and females accounted for 48.2%. The respondents included the statistical characteristics of different populations, and the samples were representative to a certain extent (Fig. 10).

## Result and discussion

### Climate change under traditional greening strategies

**Fig. 11:**
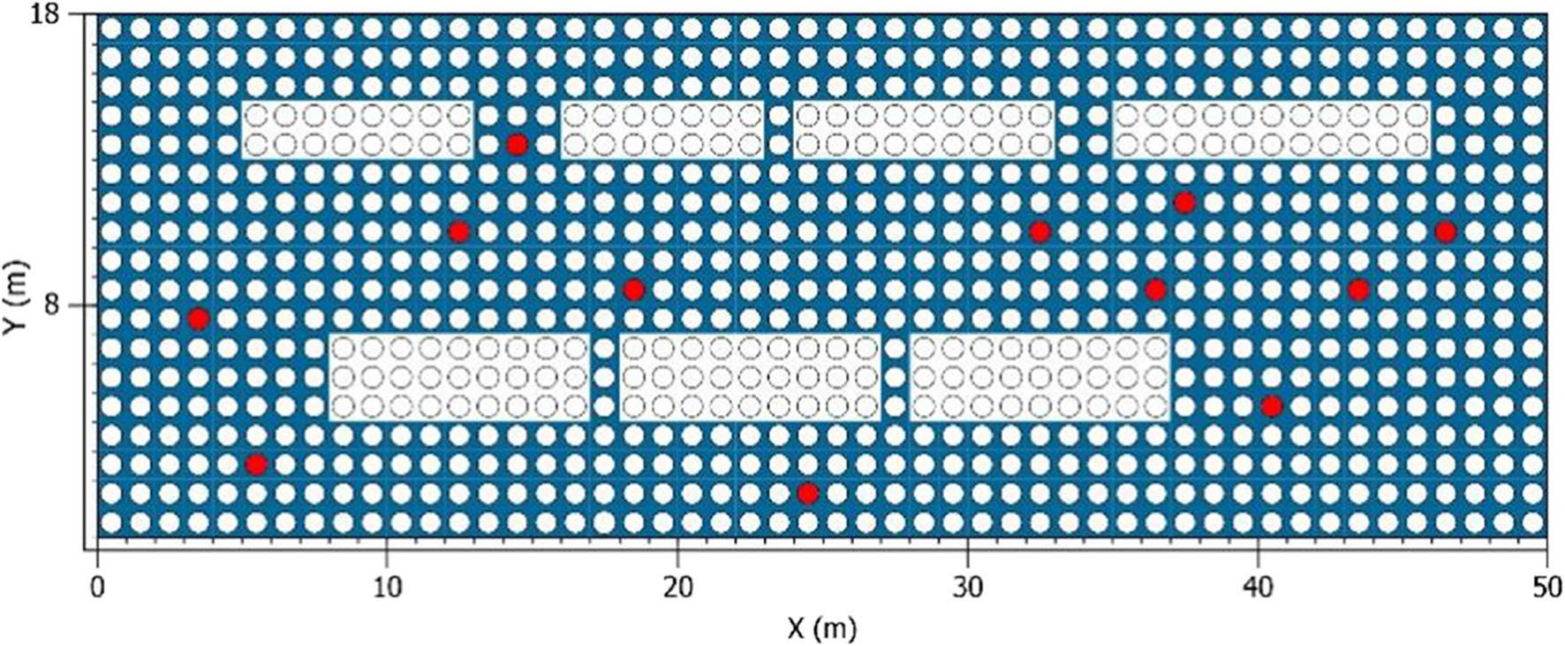
Ten planar data nodes were randomly selected

The data of 10 randomly selected points in the simulation area were collected and sorted out. In order to be more consistent with human feelings, the height of 1.5 m was selected uniformly (Fig. 11). The data node parameters are obtained by field investigation and satellite image (Table 3).

**Table 3:** Select data node parameters

Data nodes	Node coordinated (X,Y,z)	Type of underlying surface of the node
NBD1	3,7,2	vegetation
NBD2	5,2,2	Impermeable hard paving
NBD3	12,10,2	Pervious asphalt paving for
NBD4	14,13,2	highways
NBD5	18,8,2	Impermeable hard pavement
NBD6	24,1,2	vegetation
NBD7	32,10,2	Impermeable hard paving
NBD8	36,8,2	Pervious asphalt paving for
NBD9	37,11,2	highways
NBD10	40,4,2	Impermeable hard pavement
NBD11	43,8,2	vegetation
NBD12	46,10,2	vegetation
		Impermeable hard brick
		fitting
		Pervious asphalt paving for
		highways

**Fig.12:**
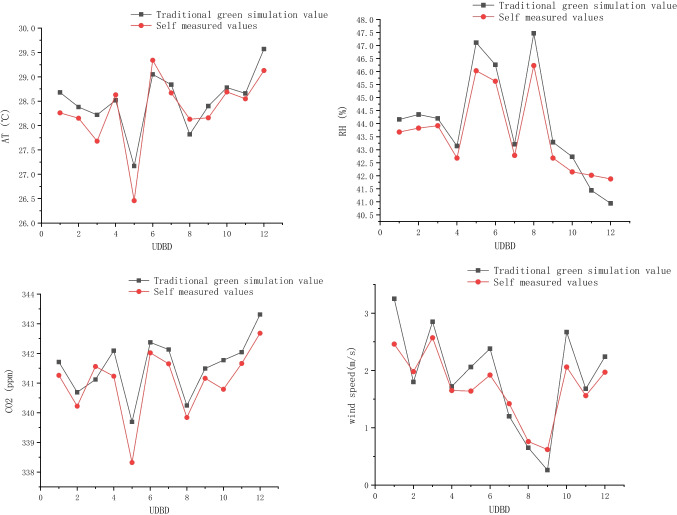
Simulation value of traditional greening. Compared with the measured values, the air temperature, humidity, CO_2_ concentration, and wind speed were obtained at different data nodes.

The extracted points are measured in real and simulated values, and the measurement results are as follows: The variation of summer air temperature of different green layouts in street showed a gradual upward trend, fluctuated from 12:00 to 14:00 at noon, and then gradually decreased from 16:00 to 17:00 after reaching the highest temperature throughout the day. The fluctuation at noon was due to cloudy conditions, which affected the temperature variation in the street. The temperature of measurement point 4 without green was higher than that of other measurement points with green throughout the day, indicating that the cooling effect of green was obvious in summer, but the temperature was slightly different under different green layouts. Measurement point 2 had only street trees in the street space, measurement point 3 had both street tree green belt and roadside green belt, and measurement point 5 was a street space composed of street tree green belt and vertical green belt. It can be seen from the figure that under the influence of the sun rising in the east and setting in the west, the measurement points 2 and 5 on the east side of the street are lower than the measurement points 3 on the west side in the morning, while under the influence of the solar radiation in the afternoon, the measurement points 2 and 5 on the east side are higher than the measurement points 3. The temperature of measuring point 2 continued to rise, reaching the highest temperature of 35.5 ℃ at 17:00, which was 2 ℃ higher than that of measuring point 3. The average daily temperature of measuring point 2 was 31.74 ℃, showing a general cooling effect. However, the temperature difference between measuring point 3 and measuring point 5 was small. The average daily temperature of measuring point 3 was 31.47 ℃, and the average daily temperature of measuring point 5 was 31.5 ℃, which was slightly higher than that of measuring point 2, and 2 ℃ lower than that of measuring point 4 without greening. In conclusion, the street space with street trees combined with rich roadside greening has the best microclimate in summer (Fig. 12).

### Climate change under three-dimensional greening strategy

**Fig. 13:**
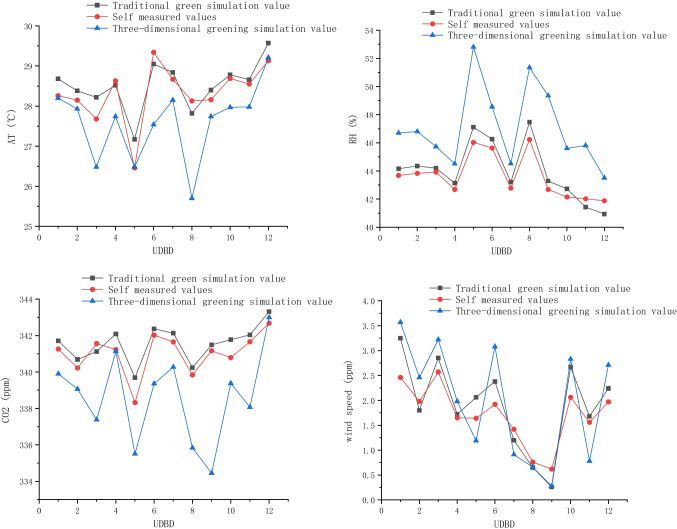
Results of air temperature, humidity, CO_2_ concentration, and wind speed under different data nodes of three-dimensional afforestation, simulated and measured values of traditional afforestation

Under the same conditions, the temperature of the green building wall was 3.5–3.6 ℃ lower than that of the control building wall, the humidity difference was 7–8%, and CO_2_ concentration decreased by about 5%. The effective radiation difference between the green surface and the non-green wall surface was 10–30%, with an average of 14.64 kcal /m^2^ hours. Roof greening enriches urban aerial landscape and increases urban green area and visible green amount. Greatly expanding the urban green space can solve the urban heat island effect. The transpiration of plant leaves can improve and reduce the temperature (Zeng 2021; Wu et al. 2019). When the urban green coverage rate is more than 40%, the improvement of temperature is more obvious. If it reaches 50%, the cool phenomenon in summer can be fundamentally changed (Fig. 13).

### Human perception of climate in stereo afforestation coverage area

In this paper, the PMV comfort index was selected to simulate the thermal comfort of the study site in summer, so as to evaluate the local geothermal environment in the study sample. The spatial distribution of PMV at 9:00, 12:00, 15:00, and 18:00 were recorded, respectively. The data indicate that the maximum PMV value is higher at 9:00, and the variation range is 0.77–2.82. The PMV in the bare area reaches 2.4, and the human body feels warm to hot, and the comfort level is low. The PMV range in the vegetated area is 0.97–1.38, and the corresponding thermal sensation is slightly warm and the comfort level is higher (Thomsit-Ireland et al. 2020). In the following hours, the PMV variation range of the study samples remained in the range of 0.2–1.3, indicating that the green space in the study sample gradually played a role, and the cool air generated by the green space and water moved to the northwest due to the effect of wind direction. At 15:00, in the 30 m buffer range of the study sample, the downwind PMV value is 0.06 smaller than that in the upwind wind; in the 280 m buffer range, the downwind PMV value (0.75) is 0.63 smaller than that in the upwind wind (1.38), and the human body can obviously feel the difference in thermal comfort.

## Discussion

### Impact of three-dimensional greening on climate change

The temperature measured data showed an overall trend of rising first and then decreasing. The temperature increased slowly between 10 AM and 12 PM, and increased rapidly between 12 and 1 PM. The temperature variation trend of the current simulation and the simulation of removing shrubs and trees was gentle, and the overall temperature first increased slowly and then decreased slowly. The setting of field monitoring sites only considered functional zones, ignoring the influence of shade on temperature. In the model, the canopy structure of vegetation was simple, and the canopy width was smaller than the actual situation (Razzaghmanesh et al. 2021), which was also one of the sources of error. The difference between simulated and measured humidity data was large, which was related to the continuous dry and windy weather days before the actual measurement. The measured and simulated values of CO_2_ concentration showed a good fitting effect, with a steady rise followed by a slow decline. The peak value of CO_2_ concentration was observed at 5 PM in field monitoring and at 4 PM in both simulations. In general, the simulated values only considered the heat exchange between atmosphere, vegetation, and buildings under ideal conditions, which could not take into account many interference factors in reality at the same time, so the overall variation trend was relatively gentle.

### Limitations of our study

Landscape planning should not only deal with aesthetics, ecology, and other issues but also deal with various new challenges brought by climate change (Liu et al. 2020). Through the research and discussion on the application of three-dimensional greening in urban architectural landscape design, it can be found that the current landscape planning system in China is not perfect (Zhi et al. 2022), and the measures to deal with climate change are mainly reflected in the small-scale landscape planning and design, which attaches importance to the adjustment and improvement of microclimate (Hai et al. 2022). However, the systematic research on the planning to deal with climate change at the macro and meso levels is still in the exploratory stage (Li and Chen 2021). It is very difficult to assess the risk in the early stage of planning, and the planning process lacks thinking about addressing climate change from an ecological perspective (Zheng et al. 2021), and pays insufficient attention to the integrity of the ecosystem. The contents and methods of planning preparation and planning implementation system need to be improved (Tao and Zi 2021; Athukorala et al. 2021).

As for this paper, there are many shortcomings in theoretical research and application practice: (1) Street landscape involves the intersection of multiple disciplines. This paper lacks a theoretical grasp of ecology, psychology, and other aspects, and the analysis is one sided. (2) As there are few application cases of three-dimensional street greening landscape in China, this paper does not put forward a comprehensive strategy for the transformation of street landscape environment based on human needs, and there are still many deficiencies in the overall study. Due to the limited data collected, I could not personally investigate and learn the domestic and foreign examples involved in this article, so I did not conduct too in-depth analysis. (3) Because Xi’an is located in northwest China, with a large geographical area and great geographical and cultural differences among cities, the implementation of three-dimensional greening needs further research. It is expected to do a comprehensive and in-depth study on the implementation and development of urban three-dimensional greening in the future.

## Conclusion

**Fig. 14:**
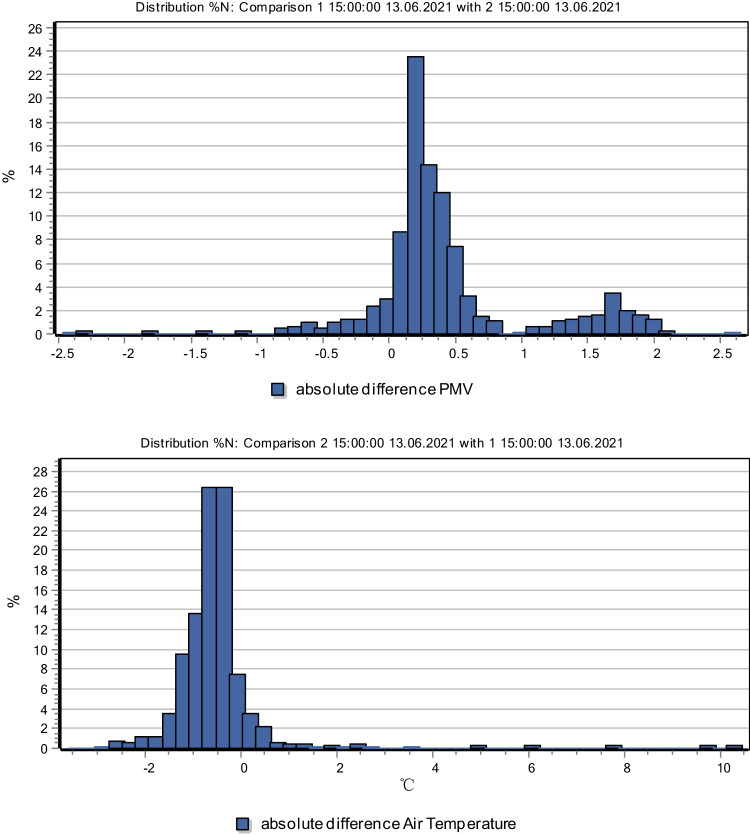
Compare the difference of air temperature between three-dimensional afforestation and traditional afforestation

**Fig. 15:**
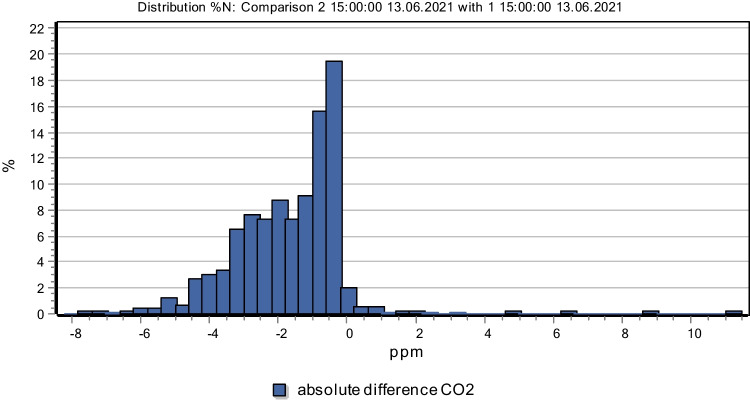
The CO_2_ concentration difference between the three-dimensional afforestation and the traditional afforestation was compared

Taking Beilin District of Xi’an as an example, this paper makes a classification investigation and technical application of its application in street, and establishes a three-dimensional green model of the sample plot in the central city of Xi’an by using local meteorological data. From the perspective of the overall improvement of urban climate, three-dimensional greening plus traditional greening is the model with the greatest environmental benefits. According to the parameter of 1.5 m from the ground in the model environment, the overall air temperature can be reduced by 0.64 ℃ on average; CO2 concentration decreased by 1.61 ppm on average. From the perspective of human perception, the PMV index increased by 0.27 on average (Fig. 14). The results show that land use and development patterns play an important role in the spatial distribution and configuration of landscape elements, and also determine the basic characteristics of landscape spatial structure, thus affecting the important role of landscape in urban development (Wellsan and Besila 2021; Qian and Qiu 2022; Morera et al. 2021). Changing land use pattern or optimizing landscape spatial structure can further improve the ability of landscape system to withstand disaster risk (Fig. 15). In cities like Xi’an, which are hot in summer, adding three-dimensional greening to traditional street greening can significantly improve the environmental microclimate, thus improving the overall urban climate.

The street landscape environment carries all the daily life of the city and creates an outdoor landscape environment suitable for people to live in. Therefore, this paper believes that the research of street three-dimensional greening landscape from the ecological perspective can continue to explore and expand the practical application of three-dimensional greening. Through the analysis of different three-dimensional greening classifications, the transformation effect of three-dimensional greening landscape on different streets can be explored. When conducting three-dimensional greening, in addition to paying attention to its ecological characteristics, it should also pay attention to its own aesthetic value, such as the plant layout principle of three-dimensional greening on the wall. It is necessary to comprehensively consider the direction of the wall, the height of the wall, the material of the wall, and the season of the plant, and combine it with the unique cultural characteristics of the local area to create a unique three-dimensional green landscape, so as to improve the image of the whole city and provide comprehensive theoretical support and application practice for the development of the three-dimensional green landscape environment. At the same time, the relevant research of this subject provides data support for the application research of three-dimensional greening in Beilin District of Xi’an, Shaanxi Province, China, and different countries, and provides new ideas for the design and research of three-dimensional urban greening in the future.

## Data Availability

All data generated or analyzed during this study are included in this manuscript.
